# Effect of Blade Geometry on γ′ Lattice Parameter and Primary Orientation of SX Cored Turbine Blades (I)

**DOI:** 10.3390/ma16010112

**Published:** 2022-12-22

**Authors:** Jacek Krawczyk, Włodzimierz Bogdanowicz, Jan Sieniawski

**Affiliations:** 1 Institute of Materials Engineering, University of Silesia in Katowice, 1a 75 Pułku Piechoty St., 41-500 Chorzów, Poland; 2 Department of Materials Science, Rzeszów University of Technology, 2 W. Pola St., 35-959 Rzeszów, Poland

**Keywords:** nickel-based superalloy, single-crystalline blades, cored turbine blades, γ′ lattice parameter, primary orientation, residual stress

## Abstract

The γ′ lattice parameter a_γ′_ and the α angle defining the primary crystal orientation of the single-crystalline cored turbine blades made of CMSX-4 superalloy were measured in the areas located near the selector situated asymmetrically, considering the top view of the blade. The distributions of the a_γ′_ and the α angle were determined along the lines parallel to the vertical blade axis Z using X-ray diffraction methods. The relations between changes in the a_γ′_(Z) and α(Z) were analyzed on the Z levels where the shape of the blade’s cross-section changes. For the first time, the local increase in a_γ′_(Z) was found near the root–airfoil connection level and near certain other root levels, which is related to the change in blade section shapes on such levels. The local extremes in α(Z), representing the dendrite bend, were observed at these levels. The increase in the a_γ′_(Z) with the local bending of dendrites was discussed concerning the local redistribution of alloying elements and local residual stresses of the γ-dendrites. For the first time, a method of analyzing the local bending of the dendrites was proposed by studying the behavior of the α(Z). The presented results concern the first stage of the research covering areas relatively close to the selector, considering the top view of the blades. The second stage will include the analysis of the areas of the blade localized at a longer distance from the selector.

## 1. Introduction

Gas turbine engines used in aerospace are among the most complex mechanical systems. The engines operate at increasingly high turbine inlet temperatures to satisfy ever-increasing working demands. Turbine blades work in temperatures very close to the melting point of the material they are made of. They are internally cooled by the air passing through intricate passages within the blade geometry to decrease the blade temperature during operation. A more complicated cooling is based on the method called “blade film cooling”, based on the supply of cooler air through small channels to the blade’s surface to eliminate direct contact with the gas flowing out of the combustion chamber [1,2].

Currently, commonly used materials are superalloys characterized by an imposing combination of high-temperature strength, phase stability, and resistance to high-temperature oxidation [3,4]. For example, their leading representative is a nickel-based second-generation CMSX-4 superalloy supplied by Cannon-Muskegon. The complex loads during the service of the blades mean that they must be resistant to high-temperature tensile strain and creep; these requirements are met by CMSX-4 superalloy [5,6,7]. The single-crystalline casts of CMSX-4 cast have high phase stability and homogeneity of the dendritic structure [8,9], as well as resistance to high-temperature corrosion [4,8].

The strength properties of particular grades of other applied superalloys, e.g., Rene supplied by General Electric, Inconel supplied by Special Metals Corporation, PWA supplied by Pratt and Whitney, and RR supplied by Rolls Royce, have been successively increased in subsequent generations.

The single-crystalline blades are usually obtained by directional crystallization using the Bridgman technique. The groups of dendrites formed during crystallization commonly create an array with a preferred crystal orientation of [001]-type, which should be parallel to the withdrawal direction [1,10].

During crystallization, various types of defects are formed, such as freckles [11,12], slivers [13,14,15], and subgrains separated by low-angle boundaries [16,17], which may negatively affect the strength parameters of the blades [18,19,20]. Misorientation of neighboring subgrains is caused by the deformation of dendrites during solidification, among other reasons [21,22,23]. Dendrite deformation results in small crystal lattice rotations and, thus, in the non-parallel growth of neighboring dendrites. Several reasons for dendrite deformation exist, for which morphological and mechanical bending can be distinguished [24]. Morphological bending is related to the changes in the local chemistry of the liquid phase influencing the growth direction of a dendrite without changing the crystal orientation of the dendrites. It is related to the so-called dendritic segregation of alloying elements [25,26,27,28,29]. If, in addition, there is a change in the crystal orientation of the growing dendrite, it is a mechanical bending related to the local distortion of the crystal lattice with a change in the lattice parameter, and hence the formation of residual stresses [30,31,32,33]. The stresses related to the dendrite bending are already created in the γ-phase and are inherited by the γ′ dendrites formed by cubic crystals. The complex geometry of the blades may also contribute to the formation of misoriented areas by bending the dendrites or areas with various primary dendrite spacing related to the material thickness [34]. In detail, other complex reasons for dendrite deformation are presented in [35].

The single-crystalline superalloys contain several alloying additives that segregate into interdendritic regions or dendrites, the arms of which may cover an area of even several hundred microns. The chemical composition heterogeneity related to dendritic segregation is disadvantageous [1,25,26,27,28,29]. Disturbances in the dendrite growth cause both local changes in the crystal orientation of dendrites and changes in their chemical composition, as well as changes in a crystal lattice parameter [36]. Such changes are disadvantageous for the blade’s strength [18,19,20] and must be reduced by a very costly homogenizing heat treatment. Local changes in the chemical composition of the blade material can be observed by measuring the lattice parameter of the γ′-phase (a_γ′_). For this purpose, the dedicated Freiberg EFG X-ray diffractometer [37] can be used, which allows for a_γ′_ measurements along the lines with the length covering a whole blade.

The cored blades are cast in ceramic molds that give them shape. The external surfaces of the blades provide the desirable aerodynamic shape, and the internal surfaces of the blades are related to the cooling bores created by ceramic cores, the shape of which is optimized for the rate of heat dissipation from different fragments of the blades. Usually, in the blades used in jet engines, the distribution of channels is very complex. Since the directional growth of dendrites is influenced by the geometry of the external and internal blades’ surfaces, the experimental differentiation of the mechanisms of the influence of the external and internal surfaces on the growth of dendrites is difficult. For the above reason, it was decided that the research presented in this paper would be carried out on the blades with a simplified shape in which the cooling bores have the simplest cylindrical form with a constant diameter, and their axis is parallel to the blade axis. It was assumed that for such blades, possible changes in the dendritic structure would be related only to abrupt changes to the outer surfaces of the blade.

The complex shape of the blades with cooling bores may create local disturbances in the dendrites’ growth [38,39,40,41]. To observe the effect of such disturbances, it is necessary to study longitudinal cross-sections of the blades parallel to the axis of the blade. Additionally, the blade axis defines the direction of the centrifugal force, which is the highest load of the blade during service, so the analysis of the lattice parameter and the primary orientation angle along this direction is essential.

Single-crystalline blade castings are commonly produced using a spiral selector [42]. In cored blades, the dendritic structure near the selector differs from that located away, as analyzed and presented in [38,43]. These differences should imply differences in the γ′-phase lattice parameter a_γ′_ and the dendrites’ primary crystal orientation described by the α angle, which is the angle measured between the crystal direction [001] and the blade axis Z. The differences suggest different mechanisms of dendrite growth. Therefore, the a_γ′_ and α angle distributions along the Z axis, in the regions relatively near the selector, considering the top view of the blades, were first investigated. The results are presented in this study. Further studies are planned to explore areas more distant from the selector, in cross-sections intersecting structures such as the cooling bore that is most distanced from the selector extension area.

The aim of this paper is to analyze the influence of the blade’s internal and external surface geometry on the γ′ lattice parameter and primary crystal orientation of single-crystalline cored turbine blades made of CMSX-4 superalloy. The current first part of the paper series concerns a longitudinal section intersecting both the selector extension area, treated as the reference area with the most negligible influence of the blade geometry on the γ′ lattice parameter and primary crystal orientation, and the nearest cooling bore. The paper is a continuation of our previous studies [38,43,44,45], which have shown that at a longer distance from the selector, considering the top view, crystal misorientation and lattice parameter inhomogeneity increase significantly, which is related to the geometry of the blade and different other parameters, e.g., a technological parameter.

## 2. Materials and Methods

The blade casts for analysis were made of CMSX-4 superalloy by the directional vertical Bridgman crystallization at a 3 mm/min withdrawal rate and with the temperature gradient in the growth chamber of G_0_ = 16 K/cm. The ALD Vacuum Technologies Co. (Hanau, Germany) VIMIC 2E–DS/SC industrial furnace, which belongs to the Research and Development Laboratory for Aerospace Materials, Rzeszów University of Technology, Rzeszów, Poland, was used to produce the blades.

**Figure 1:**
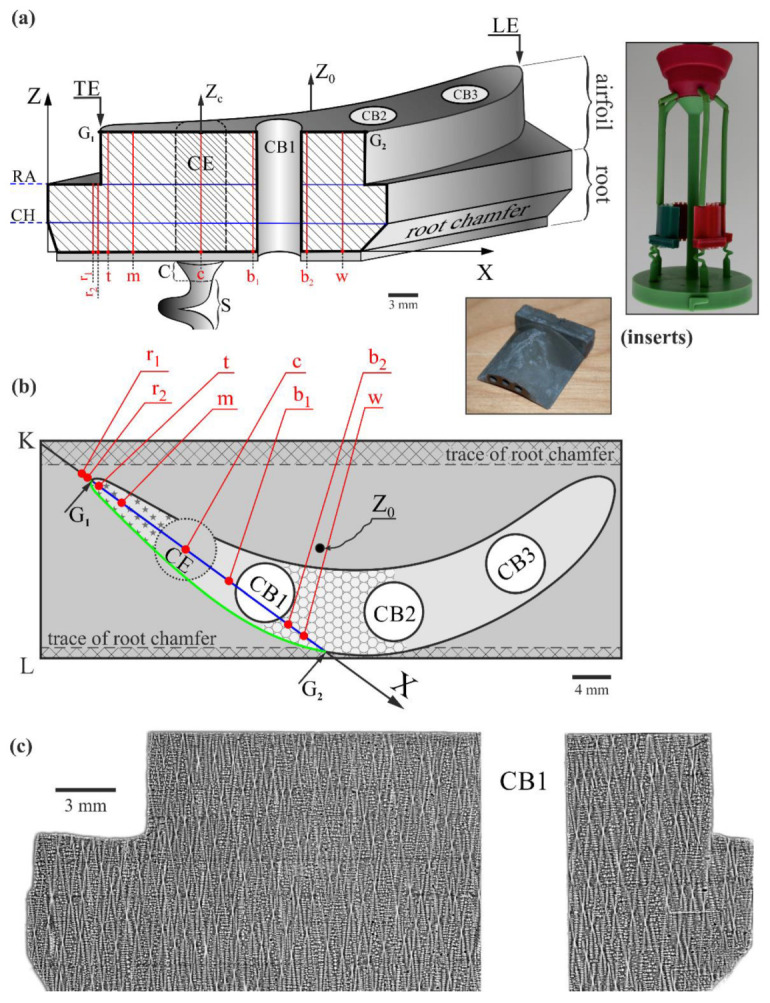
The shape of a model blade with a prepared longitudinal section (**a**) and the top view (**b**), and the dendritic structure of the analyzed section (SEM BSE) (**c**). Inserts show the wax model assembly and produced blade cast. Z_0_—main vertical axis of the blade; CH—chamfer level; RA—root–airfoil connection level; r_1_, r_2_, t, m, c, b_1_, b_2_, w—measurement lines; Z_c_—the exemplary axis of the measurement line c, the axes of the other lines have not been marked for the figure’s clarity; TE, LE—trailing and leading edges of the airfoil, respectively; S—spiral selector; C—selector continuer; CE—continuer extension; CB1, CB2, CB3—cooling bores. A description of other symbols is provided in the text.

The initially prepared wax models of the blades were placed in the wax assembly (Figure 1, insert) and coated with a ceramic layer by dipping them into a refractory slurry and coating them with refractory grains, followed by drying them to produce a ceramic mold. The total mold thickness was about 9 mm and consisted of several layers. Then the wax was removed in a steam autoclave, and the mold was fired for strength. The batch with the nominal chemical composition of CMSX-4 (wt.%: 5.6 Al, 1.0 Ti, 6.5 Ta, 6.5 Cr, 0.6 Mo, 6.0 W, 9.0 Co, 3.0 Re, 0.1 Hf, less than 0.002 C, Ni bal.) was melted using induction heating. Melting and solidification processes were carried out in a vacuum. The ceramic mold located on the chill plate was heated up to 1520 °C, and the melt of the same temperature was poured inside. When finished, the mold was removed from the furnace and separated from the blade casts (Figure 1, insert).

The blade casts contained three cylindrical cooling bores, CB1, CB2, and CB3, and a spiral selector (S) with the continuer (C) asymmetrically located relative to the main blade axis Z_0_ (Figure 1a,b). The cylindrical shape bounded by the projection of the continuer’s perimeter into the blade was named the continuer extension (CE) area. In the produced blade casts, only internal cooling was implemented as the simplest method of cooling the blades. The model blades for analysis were created from the blade casts by cutting off a fragment of the airfoil.

The model blades were intersected for the tests along the plane that was parallel to the main blade axis Z_0_ and passed through the center of the continuer extension (CE) area, the trailing edge (TE) of the airfoil (Figure 1a), and the cooling bore CB1. The exposed longitudinal section of the shape presented in Figure 1 was prepared for the tests using the standard superalloy metallographic procedure [46]. The geometry of the blade allows the distinction of two specific Z levels related to the root chamfer at the bottom of the blade limited by the chamfer level (CH-level) and the level of connection between the root and airfoil (RA-level) (Figure 1a). The levels are related to the changes in the blade transverse sections; therefore, the character of the likely changes in a_γ′_ and α has been subjected to detailed analysis.

A JSM-6480 JEOL SEM microscope (JEOL Ltd., Tokyo, Japan) was used to visualize the dendritic structure of the analyzed section surface (Figure 1c). The image was created using the backscattered electron (BSE) imaging technique by collecting several separate microimages.

The measurements of the γ′ lattice parameter and α angle defining the primary crystal orientation were completed in the Research and Development Laboratory for Aerospace Materials, Rzeszów University of Technology, Rzeszów, Poland, with the use of a dedicated EFG Freiberg Instruments X-ray diffractometer (Freiberg Instruments, Freiberg, Germany) [37]. The measurement lines parallel to the blade axis Z_0_ on the section surface are traced by a laser scanner. The values of the a_γ′_ parameter and the α angle were calculated using software with which the diffractometer is equipped, based on the Ω-scan method [37]. The method is of high sensitivity and allows the measurement of slight changes in the α angle of the order of arc minutes despite the analysis of large macroscopic surfaces [37]. The mean error of the a_γ′_ and α measurements was 0.0005 Å and 0.006°, respectively.

In the beginning, the a_γ′_ and α measurements were performed at points of line c (Figure 1a,b) located in the center of the CE area. It was assumed that the growth of dendrites in the area CE was the least disturbed by the change in the blade transverse sections. Therefore, the a_γ′_(Z_c_) and α(Z_c_) distributions could serve as reference distributions for other areas where a_γ′_ and α changes at CH and RA levels would be measurable. In the next step, measurements were performed along certain lines: m, positioned near the side surface of the airfoil and far from the side surface of the root; w, positioned near the side surface of both the airfoil and the root; r_1_ and r_2_, positioned close to TE and passing through the root only; and t, positioned close to TE and passing through both root and airfoil (Figure 1). Additionally, the measurements were performed around two lines, b_1_ and b_2_, positioned around the internal surfaces on the left and right sides of the CB1. Lines r_1_ and r_2_ covered the blade root only, and the other lines covered the root together with the airfoil. The measurement line length was several millimeters, and the a_γ′_ and α values were collected for each line in a single-pass measurement. This allowed the visualization of both small local changes and broader trends of a_γ′_(Z) and α(Z) changes occurring throughout the blade macroscopic areas.

## 3. Results and Discussion

The dendritic structure revealed in the analyzed section (Figure 1c) is typical for a longitudinal section of single-crystalline blades made of CMSX-4 superalloy using standard parameters of the technological process. The hourglass shapes visualize the oblique cuts of the dendrites.

**Figure 2:**
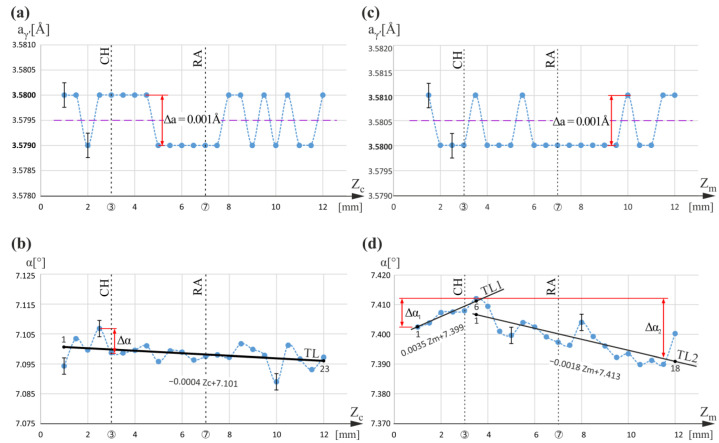
Graphs of the a_γ′_(Z_c_) and α(Z_c_) relationships obtained for measurement line c (**a**,**b**) and graphs of the a_γ′_(Z_m_) and α(Z_m_) relationships obtained for measurement line m (**c**,**d**). Z_c_ and Z_m_ are parallel to Z.

Based on our previous studies, the results of which are presented in [44], it was assumed that in the CE area (Figure 1), the growth of dendrites is steady, and the influence of the mold walls is minimized. Therefore, it was assumed that the dendrite growth inside CE proceeds without possible significant disturbances related to CH and RA levels. Hence, measurement line c, in the center of CE (Figure 1a,b), was chosen as the reference line for all studied lines, so distributions of the lattice parameter a_γ′_ and α angle of primary crystal orientation along this line were analyzed first. These distributions are shown in Figure 2a,b. The dashed lines connecting the measurement points are only drawn to visualize the fluctuations and/or increase/decrease in a_γ′_ and the α angle. They do not describe the character of the changes. It can be observed that the a_γ′_ value fluctuates around a certain level of 3.5795 Å. The a_γ′_ changes are in the range of 0.001 Å, from 3.5790 Å to 3.5800 Å over the entire measuring range Z_c_, which exceeds twice the mean measurement error (see exemplary error bars for the first and third points in Figure 2a). According to the interpretation described in [44], these a_γ′_ changes are related to the stochastic coverage by incident X-ray beam of the secondary dendrite arms’ initial or ending areas. The observed stochastic changes in the a_γ′_ in the range of 0.001 Å may be called a_γ′_ fluctuations. Because only such type changes are observed, and there were no additional changes near the CH and RA levels, it may be concluded that the fluctuations correspond to the undisturbed dendrite growth.

The α(Z_c_) changes (Figure 2b) also have only a stochastic character with values that may be approximated as a straight line TL and exceed the mean measurement error only in a few points (see exemplary error bars). No changes in α were observed near the CH (Z_c_ = 3 mm) and RA (Z_c_ = 7 mm) levels. Moreover, no correlation of α(Z_c_) with the a_γ′_(Z_c_) graph is noticeable. Therefore, it should be stated that the possible effect of a_γ′_ and α changes related to the variation of the root cross-section near the CH and RA levels is not noticeable in the CE area. Because the changes in the α angle are only stochastic, it follows that roughly in the CE area, there is no systematic bend of the dendrites near the CH and RA levels. Based on the a_γ′_(Z_c_) and α(Z_c_) graphs presented in Figure 2a,b, it can be concluded that a_γ′_ changes in the range of 3.5790–3.5800 Å are purely stochastic and correspond to dendrites formed during the steady crystallization of melt with a chemical composition strictly corresponding to CMSX-4 alloy. However, a more precise analysis of the α(Z_c_) graph shows a slightly decreasing trend in α angle. The slope of the trend line TL (points 1 to 23) is only 0.0004 [°/mm], and the maximal fluctuation of α (Δα) is about 0.01°.

Line m was selected near the side surface of the airfoil and far from the side surface of the root. Additionally, line m represented the blade area distanced both from the CE and CB1, as well as from the TE of the airfoil. The analysis of the a_γ′_(Z_m_) relationship shows only fluctuations with the value Δa_γ′_ = 0.001 Å in the range 3.5800–3.5810 Å (Figure 2c). This range is shifted towards higher values, compared to the CE area, which may indicate a slight change in the chemical composition. However, as for the CE line, only stochastic fluctuations of the lattice parameter are observed over the entire range of Z_m_ that correspond to the undisturbed dendrite growth, including the Z_m_ values around CH and RA levels.

The relationship α(Z_m_) has a trend that increases up to about Z_m_ = 3.5 mm and decreases above this value (Figure 2d). It can be assumed that there is a systematic bend of the dendrites at Z_m_ = 3.5 mm near the CH level (Z_m_ = 3 mm). Because the slope coefficient describes rates of α changes, the degree of bend can be described by the difference in the slope coefficients of the trend lines TL1 and TL2 (Figure 2d) defined for the Z_m_ < 3.5 mm and Z_m_ > 3.5 mm. The calculated coefficients are +0.0035 [°/mm] for LT1 (points 1 to 6) and −0.0018 [°/mm] for LT2 (points 1 to 18). Since the slope coefficients have opposite signs, the degree of dendrite bend can be defined as the sum of the modulus of both coefficients; i.e., for the α(Z_m_) graph, the sum (general difference) is 0.0053 [°/mm]. The range of α changes for Z_m_ < 3.5 mm is 0.008° (Δα_1_), and that for Z_m_ > 3.5 mm is 0.019° (Δα_2_), so the values are not large. As there are no systematic changes in the a_γ′_(Z_m_) on the background of Δa = 0.001 Å, it should be concluded that the observed systematic bending of the dendrites near the CH level (Z_m_ = 3mm) is not related to any measurable change in the a_γ′_ parameter. Additionally, it may be stated that neither dendrite bending nor a_γ′_ systematic changes are observed at the RA level (Z_m_ = 7 mm).

**Figure 3:**
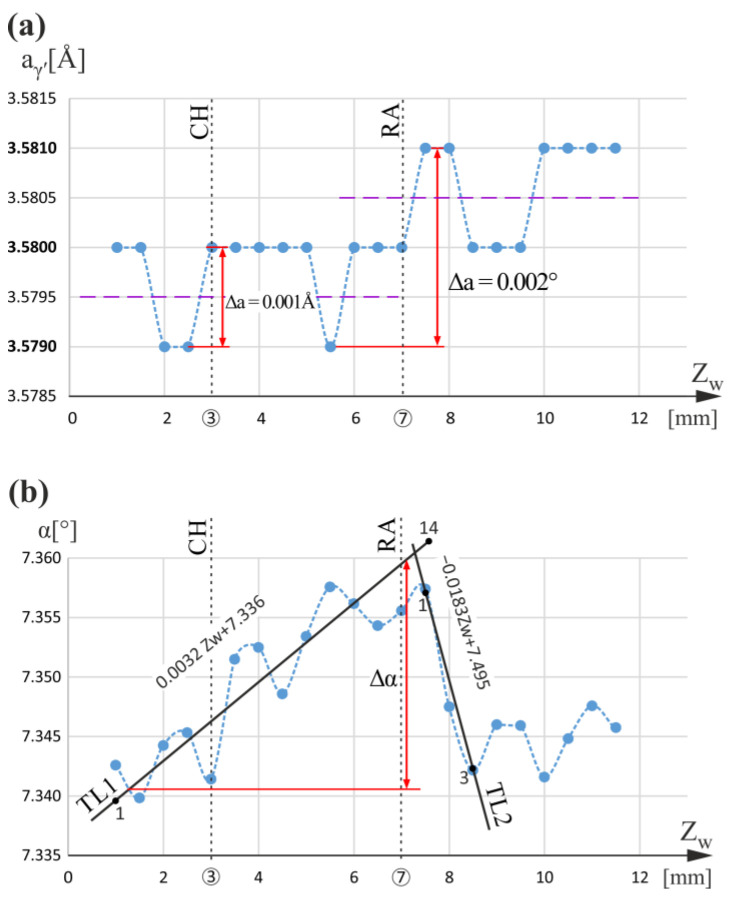
Graphs of the a_γ′_(Z_w_) (**a**) and α(Z_w_) (**b**) relationships obtained for measurement line w. Z_w_ is parallel to Z.

Next, measurements were continued for the line w positioned near the side surface of the airfoil and the root and distanced from the CE. The graphs of the a_γ′_(Z_w_) and α(Z_w_) relationship are presented in Figure 3. The dashed lines connecting the measurement points are only drawn to visualize the fluctuations and/or increase/decrease in a_γ′_ and the α angle. They do not describe the character of the changes. It was deduced from Figure 3a that for a_γ′_, there are fluctuations with the value Δa_γ′_ = 0.001 Å in the range 3.5790–3.5800 Å below the RA level (Z_w_ < 7 mm). The fluctuation range corresponds to an undisturbed dendrite array similar to that presented for the c line of the CE region. In addition, below the RA level on the α(Z_w_) graph, the systematic increase in the α value with the background fluctuations is observed, whereas, above the RA level, a similar decrease in the α value is observed. The α(Z_w_) graph analysis for line w shows a minimal effect related to the CH level of Z_w_ = 3 mm, compared to the RA level. On the other hand, the effect related to the RA level is noticeable both in the a_γ′_(Z_w_) and α(Z_w_) graphs. Slightly above the RA level, the a_γ′_ value increases to 3.5810 Å, and for the whole range Z_w_ > 7 mm, the a_γ′_ fluctuates about 3.5805 Å, which is a higher value than that for the range Z_w_ < 7 mm equal to 3.5795 Å. The trend lines TL1 and TL2 of the α(Z_w_) graph (Figure 3b) show a change in the trend from increasing (below RA level) to decreasing (above RA level). This change means that the dendrites near the RA level are highly bent. The greater degree of bending, the greater difference in the slope coefficient of the trend lines TL1 and TL2. The calculated coefficients are 0.0032 [°/mm] for TL1 (points 1 to 14) and −0.0183 [°/mm] for TL2 (points 1 to 3). Since the slope coefficients have opposite signs, the difference in the coefficients is 0.0215 [°/mm], which is significantly higher than that in the case of the line m (0.0053 [°/mm]) (Figure 2d). This means a higher bending of the dendrites near the RA level of the w line. It may be assumed that only a sufficiently large bending of the dendrites can cause observable changes in the lattice parameter.

The correlation of α change with the a_γ′_ change means that the increase in a_γ′_ may be related to the distortion of the γ′-phase’ crystal lattice due to dendrite bending near the RA level without changes in the chemical composition or to a local change in their chemical composition created during the crystallization by the redistribution of the alloying elements. The latter seems more likely for CMSX-4-type alloys containing many alloying elements. An additional argument may be the fact that the γ′-phase in the form of cubic crystals with dimensions of the order of 1 µm is formed [47] after the crystallization of γ-phase dendrites as a result of a diffusive ordering transformation. Therefore, macrostresses in bent γ-phase dendrites will not cause residual stresses of the lattice in each crystal of γ′, but rather a change in the chemical composition of following γ′ crystals. In the case of the measurement line w, the value of the α change near the RA level can be estimated as Δα = 0.018° (Figure 3b). The bending of the dendrites may be caused by local changes in the vertical temperature gradient, which may be influenced by the proximity of the external blade surfaces. The measurement line w is located in the thick fragment of the blade airfoil marked by a circle-hath in Figure 1b. The local temperature gradient in this area may be so high that the effect of a_γ′_ and α changes on the CH level is unnoticeable for the w measurement line.

**Figure 4:**
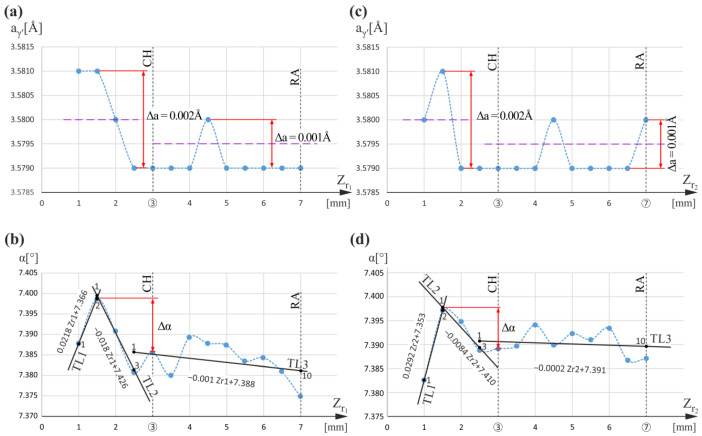
Graphs of the a_γ′_(Z_r1_) and α(Z_r1_) relationships obtained for measurement line r_1_ (**a**,**b**); graphs of the a_γ′_(Z_r2_) and α(Z_r2_) relationships obtained for measurement line r_2_ (**c**,**d**). Z_r1_ and Z_r2_ are parallel to Z.

Then, the areas on the opposite left side of the analyzed section were studied, on which measurement lines r_1_ and r_2_ were selected (Figure 1). These areas covered only the blade root, and no airfoil was above them. The measurement lines were traced at a considerable distance from the vertical side wall KL of the mold (Figure 1) so that the a_γ′_ and α measurements were not affected by additional effects related to this wall. The results are shown in Figure 4. The a_γ′_(Z_r1_) and a_γ′_(Z_r2_) graphs for both lines (Figure 4) show a maximal increase in the a_γ′_ value ranging up to 3.5810 Å for the Z_r1_ and Z_r2_ values below the CH level. The a_γ′_ changes are within 0.002 Å, and a_γ′_ value fluctuates about 3.5800 Å. The a_γ′_ changes for the Z_r1_ and Z_r2_ values observed for both r_1_ and r_2_ lines above the CH level are within 0.001 Å, and the a_γ′_ value fluctuates about 3.5795 Å. The nature of the a_γ′_(Z_r1_) and a_γ′_(Z_r2_) relationships above the CH level is similar to that observed for the line c (Figure 2a), specific for undisturbed dendrite array.

When comparing the graphs in Figure 4a,b, a correlation between the changes in a_γ′_ and α below the CH level can be observed. The increase in a_γ′_ for the r_1_ measurement line generally corresponds to changes in the slope of the TL1 and TL2 trend lines (Figure 4b), meaning dendrite bending. A similar correlation is observed for measurement line r_2_ (Figure 4d). The slope coefficients for TL1 (points 1 to 2) and TL2 (points 1 to 3) of the r_1_ measurement line are +0.0218 [°/mm] and −0.0180, respectively; hence, their difference is 0.0398 [°/mm]. The slope coefficients for TL1 (points 1 to 2) and TL2 (points 1 to 3) of the r_2_ measurement line are +0.0292 and −0.0084, respectively; hence, their difference is 0.0376 [°/mm]. These values are comparable with the value determined for the α(Z_w_) graph in Figure 3. It can also be observed that there is a gradual slight decrease in the α angle above the CH level in Figure 4b,d. The slope coefficients of the TL3 trend lines (points 1 to 10) for the α(Z_r1_) and α(Z_r2_) graphs are −0.001 [°/mm] and −0.0002 [°/mm], respectively; hence, the difference in the neighboring trend line slope coefficients (TL2) is 0.017 [°/mm] and 0.0082 [°/mm], respectively. The values suggest some bending of the dendrites. However, the difference in the slope of neighboring trend lines TL2 and TL3 is less than that calculated for TL1 and TL2. This means that, above the CH level, the dendrites are only slightly bent. In this case, in the graphs of a_γ′_(Z_r_) relationship, the fluctuation of a_γ′_ value in about 3.5795 Å is only visible, and no measurable symmetric changes in a_γ′_ are caused. Δα changes below the CH level for r_1_ and r_2_ lines range from 0.014° (Figure 4b) to 0.011° (Figure 4d), so they are significantly greater than those occurring in the CE area (0.008°, Figure 2b). Because there is a correlation between a_γ′_ changes and the degree of dendrite bending, it may be concluded that dendrite bending causes a change in a_γ′_ that may mean the presence of residual stress near the CH level.

The a_γ′_ changes can be caused by the bend of the dendrites or by local changes in heat dissipation (local changes in temperature gradient). However, it was found that with the observed slight bending of the dendrites, no changes in a_γ′_ occurred. Only at a relatively large bend, the effect of a_γ′_ change is observed, which suggests that the contribution of the alloying component segregation mechanism is controlled by the degree of dendrite bend. Therefore the cause of segregation is the bending of the dendrites. It was also found that a_γ′_ changes occur at dendrite bending levels or slightly higher Z values, i.e., in areas that crystallize later—after dendrite bending occurs. The above results in the creation of the shift of the a_γ′_ changes level in relation to the level of dendrite bending. If the cause of the segregation of alloying elements and a_γ′_ changes was a local change in the temperature gradient, this kind of shift would not occur, and a_γ′_ changes would always appear at the same level where the bending of the dendrites is visible. The above suggests that the cause of the a_γ′_ changes is the bending of the dendrites. This, in turn, may be caused by a local change in the temperature gradient or by other factors, e.g., by the influence of the mold wall [48].

**Figure 5:**
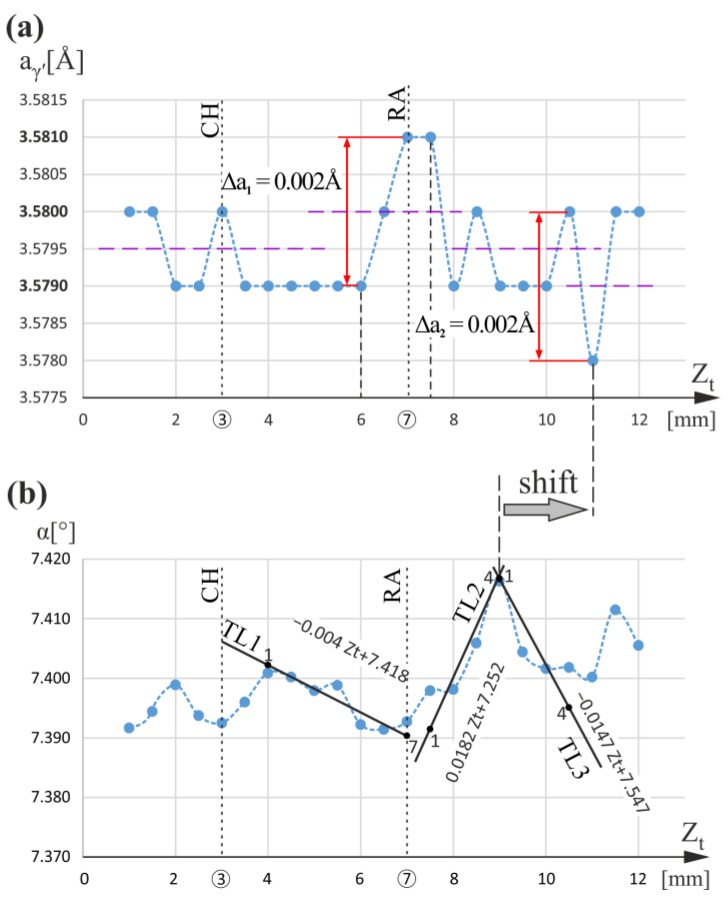
Graphs of the a_γ′_(Z_t_) (**a**) and α(Z_t_) (**b**) relationships obtained for measurement line t. Z_t_ is parallel to Z.

The measurement line t (Figure 1) traced for the entire blade height, including both root and airfoil, was chosen near the external surface of the mold close to the TE. This line is traced in the thin-walled airfoil region marked in Figure 1b by a star pattern. In addition, the measurement line t was localized close to the G_1_G_2_ line (Figure 1b), indicating a root–airfoil connection. The graphs of the α(Z_t_) and a_γ′_(Z_t_) relationships for measurement line t are presented in Figure 5. There is no similar effect for a_γ′_(Z_t_) as observed in Figure 4 below the CH level (Z_t_ < 3mm); however, the effects occurring close to the RA level (Z_t_ = 7 mm) are noticeable (Δa_γ′_ = 0.002 Å—marked as Δa_1_ in Figure 5a). The increase in a_γ′_ near the RA level with Δa_1_ = 0.002 Å is related to the bend of dendrites represented by α angle changes, which are presented by changes in the slope of TL1 and TL2 trend lines. In the α(Z_t_) graph, TL1 decreases below the RA level, and TL2 increases above the RA level. For TL1 (points 1 to 7), the slope coefficient is −0.004 [°/mm], and for TL2 (points 1 to 4), it is 0.0182 [°/mm]; hence, their difference is 0.0222 [°/mm]. For the RA level, the change in the slope coefficient sign of the α(Z_t_) trend lines and the increase in the a_γ′_ by Δa_1_ correspond to the same value of Z_t_ = 7 mm. On the other hand, the maximum in the α(Z_t_) graph occurs at Z_t_ = 9 mm, and the decrease by Δa_2_ occurs at Z_t_ = 11 mm; i.e., the positions of the extremes of a_γ′_(Z_t_) and α(Z_t_) are shifted. This means that during the crystallization, the bending of the dendrites at Z_t_ = 9 mm subsequently caused a change (decrease) in the lattice parameter of the γ′-phase, but a little later. The reduction in a_γ′_ by Δa_2_ visible in Figure 5a at the level Z_t_ = 11.0 mm may be related to the effect of dendrite “force directing” by the mold walls in the thin-walled areas of the airfoil, described in [49]. The degree of changes in the direction of the dendrite growth, i.e., their bending, is, in this case, characterized by the difference in the slope of the trend lines TL2 and TL3 (points 1 to 4) of the α(Z_t_) graph, which is 0.0329 [°/mm]. According to the interpretation presented in [45] (p. 12), such a decrease occurs when the local concentration of Re, W, and/or Mo increases, and Al, Ti, and Ta decrease. It should be emphasized that for the area below the CH level, there were no changes in a_γ′_. It can be concluded that only if a specific critical value for the bending of the dendrites is exceeded will the a_γ′_ change effect be observable.

The criterion for selecting the number of trend lines in α(z) graphs is related to the appearance of changes in a_γ′_ with a value above 0.001Å. When for some Z range there are only fluctuations a_γ′_, as is the case for 0 < Z < 7 mm in Figure 3, then only one trend line of α(Z) is selected. If there are areas with a_γ′_ changes higher than 0.001Å, that is, 0.002Å, then α(Z) should be searched for trend lines describing the bending of the dendrite (search for the α(Z) extremum). The slope coefficients of found trend lines must be higher than those at which a_γ′_ changes do not occur (e.g., as in Figure 2b,d).

**Figure 6:**
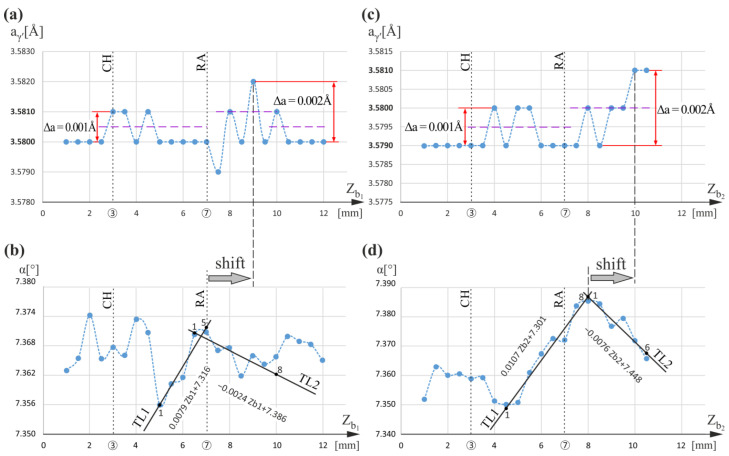
Graphs of the a_γ′_(Z_b1_) and α(Z_b1_) relationships obtained for measurement line b_1_ (**a**,**b**); graphs of the a_γ′_(Z_b2_) and α(Z_b2_) relationships obtained for measurement line b_2_ (**c**,**d**). Z_b1_ and Z_b2_ are parallel to Z.

Consecutive research was carried out for measurement lines located near and around the CB1 cooling bore. Figure 6 shows graphs of the a_γ′_(Z_b1_) and α(Z_b1_) relationships for line b_1_ located to the left of the CB1 bore, as well as the a_γ′_(Z_b2_) and α(Z_b2_) relationships for line b_2_ located to the right of the CB1 bore (Figure 1). Graphs presented in Figure 6a,c show that for both lines, above the RA level, in the range Z = 9–10 mm, the a_γ′_ value increases. Compared to the graph of a_γ′_(Z_t_) for line t (Figure 5), this effect is noticeable for the higher Z values. The graph of α(Z_b1_) (Figure 6b) shows the local decrease in α for Z_b1_ in the Z_b1_ range of 4–5 mm, i.e., above the CH level of the root, and decreasing tendency of α angle right above the RA level, i.e., above Z_b1_ = 7 mm (see local trend line TL2). Although the a_γ′_(Z_b1_) relationship in the vicinity of the CH level shows no tendency to change in a_γ′_, it shows fluctuations of a_γ′_ around 3.5805 Å with Δa_1_ = 0.001 Å. A similar character of a_γ′_(Z_b2_) relationship occurs for line b_2_ below Z_b2_ = 9 mm; however, above, the a_γ′_ increases. In the α(Z_b1_) and α(Z_b2_) graphs, the clear maxima are visible at Z_b1_ = 7 mm and Z_b2_ = 8 mm, respectively. The maximum of α(Z_b2_) is slightly shifted towards larger Z values in relation to the RA level. However, on the left and right sides of the CB1, the effect of a_γ′_(Z) changes related to the chamfering of the root is absent. It may be concluded that the influence of the blade external surfaces changing the a_γ′_ near the RA level is clearly visible, but its location is shifted toward larger Z values in relation to the dendrite bend level. This may mean that the a_γ′_ change occurred during the dendrites’ growth process, not when the dendrites were bent but slightly later.

The maxima of the α angle near the RA level, shown in Figure 6b,d, suggest large dendrite bending in this area, which can be described by the difference in the slope coefficient of the TL1 (points 1 to 5 and 1 to 8, Figure 6b and Figure 6d, respectively) and TL2 (points 1 to 8 and 1 to 6, Figure 6b and Figure 6d, respectively) trend lines. This difference is 0.0103 [°/mm] for line b_1_ and 0.0183 [°/mm] for line b_2_. Such values are greater than those for which the a_γ′_ change effect was not observed, e.g., for the line t at the CH level (Z_t_ = 3 mm). However, the a_γ′_ changes are likely to be influenced not only by dendrite bending but also by several other factors, e.g., local change in the crystallization rate of neighboring dendrites during directional growth or dendritic segregation. Since this effect does not occur with a slight dendrite bend (Figure 2 and Figure 5), it should be assumed that it is related to the degree of dendrite bending. The local increase or decrease in a_γ′_ must be related to the local redistribution of alloying elements at or near the CH and RA levels.

## 4. Conclusions

For the first time, a method of analyzing the local bending of the dendrites was proposed by studying the behavior of the α(Z). The bend is represented in the form of local extremes in α(Z).

In the continuer extension (CE) area of the blade, the local changes in the a_γ′_ lattice parameter of the γ′-phase and changes in the α angle defining the primary crystal orientation are not observed for any Z levels, especially for both the root chamfer level and the level of root–airfoil connection. This means that in the CE area, dendrites grow undisturbed under steady-state conditions directly from the continuer. The maximal stochastic change in the α angle is about 0.01°. The maximal fluctuations of a_γ′_ in the CE area are in the range of 3.579–3.580 Å, that is, 0.001 Å.

In the areas of the blade root, where the airfoil is not directly located above, the change in the root geometry below the chamfer (CH) level causes an increase in the lattice parameter a_γ′_ of 0.002 Å. The reason is the bend of the dendrites represented by a specific change in α angle with the local extremum. In turn, the reason for dendrite bending may be the changes in heat dissipation caused by the root chamfer. Local changes in a_γ′_ indicate that residual stress was generated in γ-dendrites during the crystallization, which could have been caused by local redistribution of the alloying elements. The redistribution, in turn, causes a difference in the chemical composition of γ′-phase crystals of about micrometer size along millimeter regions. An increase in a_γ′_ below the CH level should indicate a decrease in Re, W, and Mo concentrations and/or an increase in Al, Ti, and Ta concentrations.

The effect of a_γ′_ changes below the CH level does not occur in the areas where the airfoil is a continuation of the root. This may be related to the increased vertical temperature gradient, which causes masking effects of changes in heat dissipation related to the root chamfer. However, in such areas, a significant dendrite bending at the root–airfoil connection (RA) level and an increase in a_γ′_ of 0.002 Å at a higher level are observed. An increase in a_γ′_ may be related to the local redistribution of the alloying elements consisting of a decrease in Re, W, and Mo concentrations and/or the local increase in Al, Ti, and Ta concentrations. The reason for this redistribution is the earliest bending of the dendrites at the RA level.

In the areas localized around the internal surfaces of the vertical cooling bore of the blade, the a_γ′_ changes occur in the higher levels shifted from the level at which significant dendrite bending occurs. The shift may be caused by the increase in the vertical temperature gradient due to the additional heat dissipation by the ceramic mold cores forming the channels.

## Data Availability

Not applicable.

## References

[1] Dutta S., Kaur I., Singh P. (2022). Review of Film Cooling in Gas Turbines with an Emphasis on Additive Manufacturing-Based Design Evolutions. Energies.

[2] Reed R.C. (2006). The Superalloys Fundamentals and Applications.

[3] Pollock T.M., Tin S. (2006). Nickel-Based Superalloys for Advanced Turbine Engines: Chemistry, Microstructure and Properties. J. Propuls. Power.

[4] Donachie M.J., Donachie S.J. (2002). Superalloys—A Technical Guide.

[5] Zhang H., Li P., Gong X., Wang T., Li L., Liu Y., Wang Q. (2020). Tensile properties, strain rate sensitivity and failure mechanism of single crystal superalloys CMSX-4. Mater. Sci. Eng. A.

[6] Yan H., Tian S., Zhao G., Tian N., Zhang S. (2021). Creep and damage of a Re/Ru-containing single crystal nickel-based alloy at high temperature. Mater. Sci. Eng. A.

[7] Xuan W., Song G., Duan F., Xiao Z., Pan W., Zhang Y., Li C., Wang J., Ren Z. (2021). Enhanced creep properties of nickel-base single crystal superalloy CMSX-4 by high magnetic field. Mater. Sci. Eng. A.

[8] Long H., Mao S., Liu Y., Zhang Z., Han X. (2018). Microstructural and compositional design of Ni-based single crystalline superalloys—A review. J. Alloys Compd..

[9] Moreira M.F., Fantin L.B., Azevedo C.R.F. (2021). Microstructural Characterization of Ni-Base Superalloy As-Cast Single Crystal (CMSX-4). Int. J. Met..

[10] Strickland J., Nenchev B., Dong H. (2020). On Directional Dendritic Growth and Primary Spacing—A Review. Crystals.

[11] Hong J., Ma D., Wang J., Wang F., Dong A., Sun B., Bührig-Polaczek A. (2015). Geometrical effect of freckle formation on directionally solidified superalloy CM247 LC components. J. Alloys Compd..

[12] Auburtin P., Wang T., Cockcroft S.L., Mitchell A. (2000). Freckle Formation and Freckle Criterion in Superalloy Castings. Metall. Mater. Trans. B.

[13] Aveson J.W., Tennant P.A., Foss B.J., Shollock B.A., Stone H.J., D’Souza N. (2013). On the origin of sliver defects in single crystal investment castings. Acta Mater..

[14] Xu W., Wang F., Ma D., Zhu X., Li D., Bührig-Polaczek A. (2020). Sliver defect formation in single crystal Ni-based superalloy castings. Mater. Des..

[15] Sun D., Liu L., Huang T., Yang W., He C., Li Z., Zhang J., Fu H. (2019). Formation of Lateral Sliver Defects in the Platform Region of Single-Crystal Superalloy Turbine Blades. Metall. Mater. Trans. A.

[16] Strickland J., Nenchev B., Tassenberg K., Perry S., Sheppard G., Dong H., Zhang R., Burca G., D’Souza N. (2021). On the origin of mosaicity in directionally solidified Ni-base superalloys. Acta Mater..

[17] Yang W., Li J., Liu S., Wang X., Zhao J., Shi Z. (2022). Effect of Low-Angle Boundaries on the Microstructures and Tensile Properties of the Third-Generation Single-Crystal Superalloy DD9. Crystals.

[18] Rae C.M.F., Reed R.C. (2007). Primary creep in single crystal superalloys: Origins, mechanisms and effects. Acta Mater..

[19] Ram F., Li Z., Zaefferer S., Hafez Haghighat S.M., Zhu Z., Raabe D., Reed R.C. (2016). On the origin of creep dislocations in a Ni-base, single-crystal superalloy: An ECCI, EBSD, and dislocation dynamics-based study. Acta Mater..

[20] Khoei A.R., Kianezhad M. (2022). A machine learning-based atomistic-continuum multiscale technique for modeling the mechanical behavior of Ni_3_A_l_. Int. J. Mech. Sci..

[21] Aveson J.W., Reinhart G., Nguyen-Thi H., Mangelinck-Noël N., Tandjaoui A., Billia B., Goodwin K., Lafford T.A., Baruchel J., Stone H.J., Huron E.S., Reed R.C., Hardy M.C., Mills M.J., Montero R.E., Portella P.D., Telesman J. (2012). Dendrite bending during directional solidification. Superalloys 2012.

[22] Aveson J.W., Reinhart G., Nguyen-Thi H., Mangelinck-Noël N., D’Souza N., Stone H.J. Origins of misorientation defects in single crystal castings: A time resolved in situ synchrotron X-ray radiography study. Proceedings of the MATEC Web of Conferences 14, 2nd European Symposium on Superalloys and their Applications.

[23] Mullis A.M., Walker D.J., Battersby S.E., Cochrane R.F. (2001). Deformation of dendrites by fluid flow during rapid solidification. Mater. Sci. Eng. A.

[24] Doherty R.D. (2003). Comments on Mechanical deformation of dendrites by fluid flow during the solidification of undercooled melts. Scr. Mater..

[25] Cheng K.Y., Jo C.Y., Kim D.H., Jin T., Hu Z.Q. (2009). Influence of local chemical segregation on the γ′ directional coarsening behavior in single crystal superalloy CMSX-4. Mater. Charact..

[26] Warnken N. (2016). Studies on the Solidification Path of Single Crystal Superalloys. J. Phase Equilibria Diffus..

[27] Seo S.M., Jeong H.W., Ahn Y.K., Yun D.W., Lee J.H., Yoo Y.S. (2014). A comparative study of quantitative microsegregation analyses performed during the solidification of the Ni-base superalloy CMSX-10. Mater. Charact..

[28] Ber L.B., Rogozhkin S.V., Khomich A.A., Zaluzhnyi A.G. (2022). Distribution of Alloying Element Atoms between γ- and γ′-Phase Particles in a Heat-Resistant Nickel Alloy. Phys. Met. Metall..

[29] Zhang J., Zong H., Lu F., Huang T., Wang D., Zhang J., Zhang J., Su H., Liu L. (2022). Synergistic effects of Re and Ta on the distribution of W in Ni-based superalloys. Intermetalics.

[30] Aveson J.W., Reinhart G., Goddard C.J.L., Nguyen-Thi H., Mangelinck-Noël N., Tandjaoui A., Davenport J.R., Warnken N., Di Gioacchino F., Lafford T.A. (2019). On the Deformation of Dendrites During Directional Solidification of a Nickel-Based Superalloy. Metall. Mater. Trans. A.

[31] Lee D.N., Kim K.H., Lee Y.G., Choi C.H. (1997). Factors determining crystal orientation of dendritic growth during solidification. Mater. Chem. Phys..

[32] Dragnevski K., Mullis A.M., Walker D.J., Cochrane R.F. (2002). Mechanical deformation of dendrites by fluid flow during the solidification of undercooled melts. Acta Mater..

[33] Hallensleben P., Scholz F., Thome P., Schaar H., Steinbach I., Eggeler G., Frenzel J. (2019). On Crystal Mosaicity in Single Crystal Ni-Based Superalloys. Crystals.

[34] Körber S., Fleck M., Völkl R., Glatzel U. (2022). Anisotropic Growth of the Primary Dendrite Arms in a Single-Crystal Thin-Walled Nickel-Based Superalloy. Adv. Eng. Mater..

[35] Scholz F., Cevik M., Hallensleben P., Thome P., Eggeler G., Frenzel J. (2021). A 3D Analysis of Dendritic Solidification and Mosaicity in Ni-Based Single Crystal Superalloys. Materials.

[36] Durand M., Cormier J., Paumier F., Katnagallu S., Saksena A., Kontis P., Pettinari-Sturmel F., Hantcherli M., Franchet J.M., Dumont C. (2022). Chemical redistribution and change in crystal lattice parameters during stress relaxation annealing of the AD730™ superalloy. Acta Mater..

[37] Berger H., Bradaczek H.A., Bradaczek H. (2008). Omega-Scan: An X-ray tool for the characterization of crystal properties. J. Mater. Sci. Mater. Electron..

[38] Krawczyk J., Bogdanowicz W., Sieniawski J. (2021). The Influence of the Cooling Bores on Crystal Orientation and Lattice Parameter in Single-Crystalline Cored Turbine Blades. Materials.

[39] Yan X., Zhang K., Deng Y., Sun R., Lin L., Zhang X. (2014). The effects of DS blade’s geometry features on material’s creep strength. Propuls. Power Res..

[40] Szeliga D. (2018). Effect of Processing Parameters and Shape of Blade on the Solidification of Single-Crystal CMSX-4 Ni-Based Superalloy. Metall. Mater. Trans. B.

[41] Chen C., Sun J., Diao A., Yang Y., Li J., Zhou Y. (2022). On the dendrite deformation and evolution mechanism of Ni-based superalloy during directional solidification. J. Alloys Compd..

[42] Wang B., Zeng L., Ren N., Xia M., Li J. (2022). A comprehensive understanding of grain selection in spiral grain selector during directional solidification. J. Mater. Sci. Technol..

[43] Paszkowski R., Bogdanowicz W., Szeliga D. (2021). The Low-Angle Boundaries Misorientation and Lattice Parameter Changes in the Root of Single-Crystalline CMSX-4 Superalloy Blades. Materials.

[44] Krawczyk J., Bogdanowicz W. (2022). Correlation between the Dendritic Structure and Lattice Parameter of γ′-Phase in Single-Crystalline Turbine Blades Made of Superalloys. Materials.

[45] Krawczyk J., Bogdanowicz W. (2021). The Influence of the Cooling Bores on the Dendritic Structure and Crystal Orientation in Single-Crystalline Cored CMSX-4 Turbine Blades. Materials.

[46] Zipperian D.C. (2011). Metallographic Handbook.

[47] Krawczyk J., Paszkowski R., Bogdanowicz W., Hanc-Kuczkowska A., Sieniawski J., Terlecki B. (2019). Defect Creation in the Root of Single-Crystalline Turbine Blades Made of Ni-Based Superalloy. Materials.

[48] Bogdanowicz W., Krawczyk J., Tondos A., Sieniawski J. (2017). Subgrain boundaries in single crystal blade airfoil of aircraft engine. Cryst. Res. Technol..

[49] Bogdanowicz W., Krawczyk J., Paszkowski R., Sieniawski J. (2019). Primary Crystal Orientation of the Thin-Walled Area of Single-Crystalline Turbine Blade Airfoils. Materials.

